# How to Study Chronic Diseases—Implications of the Convention on the Rights of Persons with Disabilities for Research Designs

**DOI:** 10.3389/fpubh.2017.00088

**Published:** 2017-05-03

**Authors:** Sebastian von Peter, Patrick Bieler

**Affiliations:** ^1^Psychiatric University Clinic of the Charité, St. Hedwig Hospital, Berlin, Germany; ^2^Institute of European Ethnology, Humboldt University of Berlin, Berlin, Germany

**Keywords:** method, disability, practice, relationality, context

## Abstract

**Background:**

The Convention on the Rights of Persons with Disabilities (CRPD) has been received considerable attention internationally.

**Methods:**

The Convention’s main arguments are conceptually analyzed. Implications for the development of research designs are elaborated upon.

**Results:**

The Convention entails both a human rights and a sociopolitical dimension. Advancing a relational notion of disability, it enters a rather foreign terrain to medical sciences. Research designs have to be changed accordingly.

**Conclusion:**

Research designs in accordance with the CRPD should employ and further develop context-sensitive research strategies and interdisciplinary collaboration. Complex designs that allow for a relational analysis of personalized effects have to be established and evaluated, thereby systematically integrating qualitative methods.

## Introduction

The Convention on the Rights of Persons with Disabilities (CRPD) has been adopted by the UN 2006 and, since then, received considerable attention internationally. As of December 2016, it held 172 signatories and 160 parties, a lot more than the UN Declaration of Human Rights had gathered in more than 60 years of history (1, 2).

Such a proliferation seems to be due to the Convention’s obvious appeal to practice, maybe caused by the fact that for the first time NGOs have participated in the formulation of a human rights instrument. It, further, can be explained by its wide area of application: referring to any form of “long-term physical, mental, intellectual, or sensory impairments,” it applies to more or less every chronic condition within the field of medicine (3).

Given this widespread attention, this article will explore the CRPDs implications for developing research designs. First, the Convention’s historical roots will be briefly analyzed, laying the grounds for a conceptual analysis of its two main argumentative strands. Then, the Convention’s relational notion of disability will be focused upon, as it is thought to be a fundamental shift to previous approaches. Finally, five adjustments to conventional randomized controlled trial (RCT) research models will be proposed that have the potential to take the Convention’s relational notion of disability into account.

## The Convention’s Relational Notion of Disability

The CRPD covers an array of various aspects that cannot be fully considered here due to shortage of space. This may also not be necessary, as these numerous aspects can be summed up into two main argumentative strands that are to be found in Article I, the treaty’s preamble and description of purposes.

*First*, the CRPD follows a *human rights approach*: it advances the rights of persons with disabilities, promoting their claims for full participation in education, mobility, health, employment, and in daily and political life. Drawing on activist ideas of the 1960s feminist and minority rights movements, the CRPD thus serves as another catalyst within the longstanding tradition of human rights-focused approaches within the field of medicine. This human rights aspect is not further elaborated upon in this article. Yet, it should be mentioned briefly that it suggests the use of participatory research strategies (4): persons with impairments have to be systematically integrated within the development of research designs, data collection and interpretation, and dissemination of results (5). This has to be, as to the Convention’s legislation. And it would be wise to do, as these persons know best how to struggle along within life so as to come to terms with (their) impairments.

*Second*, the CRPD contains a *sociopolitical dimension*, proposing a relational notion of disability that acknowledges both the physical and societal barriers preventing disabled persons’ participation in society. It is here, as will be argued forthwith, where the Convention enters a rather foreign terrain to medical researchers and practitioners. Origins for such a relational notion can be found in the “social model of disability” that has been advanced in the late 1980s by critical, post-structuralist activists to react to the so felt dominance of a deficit orientated, mainly medically based notion of disability (6). In lieu of understanding disabilities to be grounded in individual impairments, they have been perceived to result from systemic barriers and exclusive attitudes by society, thus being an effect, or better artifact of processes of social constructions (7).

**Figure 1:**
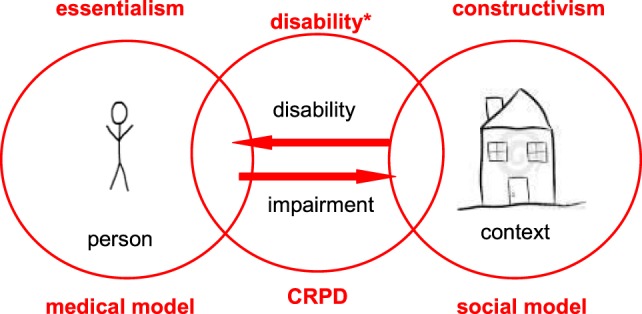
**The locus of disability varies: whereas the medical model primarily holds individual impairments’ responsible, the social model of disability rather focuses on contextual constraints**. The Convention on the Rights of Persons with Disabilities (CRPD) combines both models in taking into account both a person’s impairments and factors in his or her environment that only in relation hinder their participation in society.

Yet, in contrast to such a rather constructivist model of disability, the CRPD also takes the impact of impairments into account. As per the Convention’s first article, disabilities*^1^ result from “impairments that *in interaction* with various barriers may hinder their [persons’] full and effective participation in society on an equal basis with others” (3). Hence, a relational notion of disability* is used, reciprocally connecting individual deficits to contextual constraints: neither any impairment nor an environment alone are perceived to be disabling *per se*, but only the combination, or better mismatch of both, impeding a person’s participation in society (Figure 1).

## Research Designs in Accordance with CRPD

Such a relational, context-dependent definition of disability* seems to be rather alien to the field of medicine. This has mostly historical reasons, whose closer examination would exceed the limits of this article: in short, within the medical field, (chronic) diseases have traditionally been perceived to be stable and unchangeable traits across situations that exert their disabling effects regardless of any interdependence with their context (8). Accordingly, RCT research designs have been developed that allow for investigating the outcome(s) of an intervention under “ideal conditions,” i.e., devoid of contextual interferences: context is either deliberately neutralized or reduced to a well defined, hence controllable number of fixed variables that ideally function mutually exclusively (9).

Randomized controlled trials certainly are the most systematic way to evaluate the effects of an intervention: by disentangling an object of analysis from its context, they are able to precisely demarcate a therapy’s specific outcomes. Yet, taking the Convention’s relational notion of disability* seriously might require adaptations to such proceeding: Disabilities* emerging from the interplay between individual impairments and contextual conditions ask for interventions that are equally complex and whose specific effects might not be as clearly delineated. What is more, actively neutralizing the context of an intervention might be problematic, as context, according to the Convention’s definition, might carry aptitudes for change.

Thus, five adjustments to conventional RCT research models will be proposed in the following some of them being acknowledged others proposing rather novel ideas to adapt research designs to the Convention’s relational model of disability*. In order to illustrate these adjustments, and also to highlight that research designs for acute disorders might considerably differ, concrete examples will be presented that employ globally prevalent chronic conditions out of various medical fields.

### Context Sensitivity

Taking the Convention’s relational notion of disability* seriously prompts to focus an intervention’s context in more diligent ways than traditional RCT designs allow for. Disability* resulting from reciprocal effects between both an impairment and a disabling context, the latter is not only considered a disturbance but also to actively contribute en- or disabling a person. A stressful environment, for instance, might simply be seen as a confounding variable when intervening with stenocardia (10)—or it might be perceived to be an active component itself reducing an impaired person’s chances for participation. Both cases highly differ in how they acknowledge contextual influences, the former reducing context to a set of avoidable and, at best, clearly circumscribable variables, the latter rendering it to an active and complex ingredient in its own right.

Thus, research designs in accordance with the Convention’s relational notion of disability* should employ context-sensitive strategies: testing an intervention against various contexts has been advocated to identify its active ingredients (11). Randomizing groups of subjects instead of individuals (12) or evaluating an intervention’s effects in relation to various fields (13) equally serve to systematically investigate the extent to which contexts may enable or constrain change. And, finally, ethnographic methods may be used to identify contextual active ingredients, as further elaborated below.

### Interdisciplinary Collaboration

If context counts as constrain or resource that dis-/enables change, research designs, further, should dedicate more attention to these transformative potentials. This seems to be particularly reasonable in the case of chronic conditions—when cure of impairments can often not be hoped for. Contextual constraints or resources may involve norms, moral judgments, meta-level policies, or regulations. They might be of technical or social nature, potentially residing in an intervention’s material environment or in an organization’s social architecture. To specify them helps to fill the usual “black box” between environmental conditions and the efficacy of an intervention and thereby to “ecologically model” approaches of care (14).

Yet, context is polyvalent. Various expertise and knowledge thus are required to understand a context’s transformative effects. A good example is the interdisciplinary research collaborations that have been stimulated by the after war need for extensive rehabilitation and that have been labeled “Universal Design” later on in history: these collaborations joined various techniques and instruments to develop, evaluate, and promote accessible design mainly for persons with physical disabilities (15). This is one of only few examples for systematic interdisciplinary inquiry in the field of chronic illness. Usually the disciplinary ethos not only in medical sciences is highly compartmentalized and there is a shortage of adequate models for how to initiate and pursue interdisciplinary collaboration (16).

### Relational Analyses

And yet, what determines a disability* most: a person’s impairments or his or her surrounding? This question asks to decide either for a medical or a social model of disability*. In contrast, the CRPD takes a step forward in locating disability* *in between*, i.e., as an intermediate function of interrelated elements of both individual and contextual conditions. Accordingly, analyzing the effects of an intervention should focus on *relations*, i.e., the analysis of the complex interplay between both a person and his or her surrounding, without methodically granting privilege to one of each. For instance, when measuring the effects of some assistant technology for persons with diabetes (insulin injection or pump, blood sugar meter, etc.), it might be difficult to divide out the complex and mutual interrelations between person and technology, requiring methods that allow for analyzing their effects together (17).

It is also in this respect that research designs are underdeveloped: relational forms of analyses are rare to inexistent in the field of medicine. Most notably, there is a lack of theories how conceptualize change models relationally. Exceptions are ethnographic methods, and in particular the longstanding method of participant observation that allows for reciprocally analyzing the mutual effects between an intervention and a particular situation (9, 18). Further, practice theories, originating in various social sciences (19, 20), have proven to be useful, also in researching disabilities* (21, 22), for understanding interrelations between both an impaired person and his or her environment.

### Web of Effects

In the everyday treatment of chronic diseases, complex interventions are frequently used (23): these forms of care produce change in rather non-linear ways; they usually involve multi-composite arrays of various and mostly highly interlinked, active ingredients. To give an example various psychosocial therapies for psychotic disorders can be perceived to be complex interventions: the critical ingredients of assertive community treatments have been analyzed (24), the active components of peer-support delineated (25). In both examples, change models have been developed that point to often highly complex, reciprocal relationships between interventions, context, and outcomes.

Thus, interventions for chronic diseases often impact due to a non-linear dynamics of manifold interacting components. Accordingly, and in reference to the venerable model “web of causation” (26), a *web of effects* has to be conceptualized: research designs in accordance with the Convention’s relational notion of disability* should attempt to analyze a highly interrelated range of primary, secondary, process-related, intermediary etc outcomes. They should be well equipped to bring forth complexity, instead of generating reduced findings. This, above all, requires systematic process evaluation (27): it has to be clear, *how, why*, and *where* an intervention may produce change. This requires the systematic integration of qualitative methods, an argument that will be further elaborated upon in the next section.

### Personal(ized) Outcomes

As shown by the concepts and results of Personalized Medicine approaches, individuals are (biologically and socially) diverse (28): they may, in response to different contexts, react differently to an intervention, as demonstrated for various cancer treatments (29). Or persons may employ an intervention for various goods, making use of it in personal ways: for instance, Beta blockers are used by patients with hypertension not only to control their blood pressure but also for sedative effects. In the same way, a range of ethnographies illustrate how persons with chronic diseases creatively act upon a particular intervention: engaged in sustained efforts, they calibrate it until it (usually temporarily) fits—not only to the persons’ own prospects but also to the context around them (19).

Thus, an intervention might have various effects: it may be received differently or be used for different purposes. As a result, it might be inappropriate to come up with short term proceedings or interventionist ready-mades. Instead, evaluating an interventions effects involves a primarily investigative approach, exploring the multiple and often highly individual ways in which it fits to a persons condition or has been tailored to his or her situation (30). Merely employing patient related outcome measures does not suffice in this context. Instead, qualitative methods should be integrated as they allow for analyzing the full range of (experienced, potentially variable, and context-dependent) effects that might be overlooked, when focusing only on a fixed set of (quantitatively measurable) outcomes.

## Conclusion

Randomized controlled research designs are able to reduce the effects of contextual interferences. That is their strength, yet, in the light of the CRPD, also their weakness. Research designs in accordance with the Convention’s relational notion of disability*, should employ context-sensitive research strategies. They have to apply and further develop models and concepts for interdisciplinary collaboration. Third, designs that allow for relational analyses, to date rather foreign to the medical field, would be helpful to capture relational modes of change. A web of effects has to be conceptualized, instead of focusing a reduced set of outcomes. For this purpose and for the analysis of personalized effects, qualitative methods have to be systematically integrated.

## Author Contributions

SP had the idea for the outline of the paper; further, he wrote the article. PB contributed in precisely exploring the relational notion of disability, thereby extensively drawing on his social scientific background.

## Conflict of Interest Statement

The authors declare that there are no conflicts of interest. This research received no specific grant from any funding agency in the public, commercial, or not-for-profit sectors.

## Footnotes

^1^As mentioned above, the rather constructivist notion of “disability,” used by scholars of the Disability Studies, differs from the Convention’s notion of disability. Due to this shortage of terminology, in the following, disability will be used to frame the Convention’s dynamic and relational version of it.
